# Non-hexagonal neural dynamics in vowel space

**DOI:** 10.3934/Neuroscience.2020015

**Published:** 2020-08-04

**Authors:** Zeynep Kaya, Mohammadreza Soltanipour, Alessandro Treves

**Affiliations:** 1 SISSA–Cognitive Neuroscience, via Bonomea 265, 34136 Trieste, Italy; 2 NTNU–Centre for Neural Computation, Trondheim, Norway

**Keywords:** grid cells, vowel space, formants, hexagonal symmetry, EEG, diphthongs

## Abstract

Are the grid cells discovered in rodents relevant to human cognition? Following up on two seminal studies by others, we aimed to check whether an approximate 6-fold, grid-like symmetry shows up in the cortical activity of humans who “navigate” between vowels, given that vowel space can be approximated with a continuous trapezoidal 2D manifold, spanned by the first and second formant frequencies. We created 30 vowel trajectories in the assumedly flat central portion of the trapezoid. Each of these trajectories had a duration of 240 milliseconds, with a steady start and end point on the perimeter of a “wheel”. We hypothesized that if the neural representation of this “box” is similar to that of rodent grid units, there should be an at least partial hexagonal (6-fold) symmetry in the EEG response of participants who navigate it. We have not found any dominant n-fold symmetry, however, but instead, using PCAs, we find indications that the vowel representation may reflect phonetic features, as positioned on the vowel manifold. The suggestion, therefore, is that vowels are encoded in relation to their salient sensory-perceptual variables, and are not assigned to arbitrary grid-like abstract maps. Finally, we explored the relationship between the first PCA eigenvector and putative vowel attractors for native Italian speakers, who served as the subjects in our study.

## 1. Introduction

How can the human brain perceive and process fleeting speech signals and map them into linguistic representations? At the lowest level, this requires mapping acoustic signals into sound categories, the phonemes, a highly non-trivial process, despite the ease with which we execute it continuously, and thus distinguish first syllables and then words from each other. Phonemes are classified either as vowels or consonants according to their spectral properties. Different consonants are produced by setting a variety of vocal tract parameters, which interact with each other and with those of adjacent phonemes, resulting in an acoustic signal that is difficult to decode by eye, looking at the spectrogram [1]. Different vowels, in contrast, are produced primarily by opening more or less the vocal tract, and shifting the articulation of the tongue, which modulates the opening, from the back to the front, resulting in an acoustic signal that is, to a reasonable approximation, just a point in a two-dimensional space. Different vowels occupy, for any given speaker, distinct portions of this space and the listener can readily decode them, in most cases, by locating on this 2D space the sound just heard. This raises the issue of whether to “navigate” such space, or just to orient in it, we use similar mechanisms to those observed in the spatial representations of rodents.

Two seminal studies have in fact advanced the idea that the grid cells discovered in rodents [2] may be relevant to human cognition, by first observing an approximate 6-fold symmetry with respect to direction of motion, interpreted to be of grid-cell origin, in the BOLD signal of human subjects moving in a virtual reality arena [3]; and then observing it, again in several cortical areas, in subjects “moving” in the abstract 2D continuum of drawings of a bird with extendable legs and neck [4].

### 1.1. Vowel space

Frequency analyses of a vowel sound show a clear harmonic spectrum, determined by the vibration of the vocal folds, with several components, each with its own amplitude and bandwidth. The first one is the pitch (fundamental frequency, or F0), which varies from individual to individual, and is markedly influenced e.g. by gender. Female speakers usually have higher pitch than male speakers. Which among the higher frequencies has significant power determines vowel identity, as the speaker adjusts the vocal tract to make specific harmonics resonate. In particular, the first two resonant frequencies, labelled F1 and F2, in many languages usually suffice to define which vowel is being heard, at least after a brief adaptation to a specific speaker. Some vowel contrasts, in some languages, require analysis of additional components, or of how the frequency of the first two components varies in time. With these qualifications, still it is remarkable to what extent vowel space can be regarded as a plane, in which the two dimensions simply reflect the extent to which the vocal tract is open (F1), and where from the back to the front is the tongue restricting the opening (F2). The most appropriate analogy is actually to a trapezoidal box, since F1 and F2 are limited by boundaries that are speaker-dependent, but which can be roughly described by 4 lines, for example 200 Hz < F1 < 900 Hz, and 300 Hz + (2/3) × F1 < F2 < 3000 Hz – (4/3) × F1. Understanding speech therefore involves, *inter alia*, localizing speakers within their specific vowel trapezoid, several times per second, when they utter a vowel. Further, if the sound is not approximately point-like, as for an isolated vowel, but rather a short trajectory, as for a diphthong, the navigation metaphor takes an even more concrete poignancy [5].

**Figure 1.**
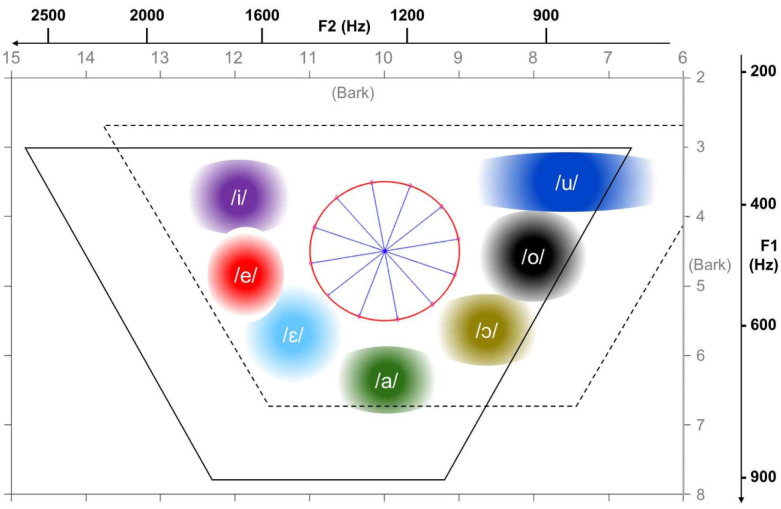
Vowel space. The (F1, F2) axes indicate both Hertz and Bark scales–notice their directions. Conservative estimates of the typical positions of 7 standard Italian vowels are reproduced from [7], though usually female speakers reach substantially higher frequencies for front and open vowels (solid trapezoid) and male speakers lower F2 for back vowels (dashed trapezoid). The stimuli used in the experiment were sliding along the rays of the wheel at the center.

The openness of the vocal tract and the location of the tongue, hence of the main restriction, are expressed by the first and second formant frequencies F1 and F2 in a strikingly direct fashion, which allows us to suspend judgment on whether acoustic features or motor parameters are more important, since in this case they largely coincide [6], There is only a non-linearity, such that at low frequencies the minimum discriminable threshold tends to be constant, ΔF, whereas at high frequencies the relative threshold ΔF/F tends to be constant. This non-linearity is usually captured by charting the vowel plane not in Hertz but in Barks, a unit defined in a number of nearly equivalent ways, linearly related to Hertz at low frequencies and logarithmically at higher frequencies, as shown in Figure 1. Standard vowels in Italian cluster along the two oblique and the short bottom border of the trapezoid, leaving largely empty, both for female and male speakers, the area close to the top border (where other languages, instead, have several standard vowels, e.g. Turkish). We have attempted to exploit this peculiarity in the design of our experiment.

We intentionally overlook here other features that can help classify vowels, such as rounding or duration, which are less important in Italian. Characterizing and contrasting the role of rounding in Turkish and in Italian has been the focus of a parallel study in our group, to be published elsewhere. We note in passing that the limited relevance of rounding and other variables, in Italian, reduces the likely importance of top-down modulation for phoneme identification, making localization on the 2D plane largely sufficient (cp [8] vs [9]). Approximately point-like vowels are also called monophthongs. If the tongue moves during vowel production from one position towards another, it results in the production of one syllable with two fused vowels, which are together called a diphthong (“two sounds” in Greek). Note, however, that what is a diphthong and whether its realization is one single phoneme or two adjacent phonemes is language dependent, and ultimately up to the listener [10]. For example, /ai/ can be recognized as a diphthong in a given language, but not its reverse, i.e. /ia/. Further, many educated Italians will find it unacceptable to break e.g. /iε/ into two syllables in certain words of particular etymology, like *siero*, which younger Italians often break, oblivious of its origin from the Latin *se˘rum* [11]. In many languages, it is conventionally assumed that some diphthongs carry their own phoneme identity, distinct from that of the combination of their constituents. Observing their spectrograms, one sees that diphthongs have at least one portion with largely steady (F1, F2) values, and usually two, at the beginning and the end, and one transition portion in the middle, when the two resonances smoothly shift from one set of values to another [12]. On the 2D plane in Figure 1, they can be represented as trajectories. The slope and the duration of the transitioning part can be discriminative perceptual cues [13], but we consider a subset of quasi-diphthongs that have slope and duration, and hence distance covered in the (F1, F2) Bark plane, constant.

In analogy with spatial navigation, therefore, during which neuronal activity patterns are thought and sometimes observed to settle into stable states of activity that correspond to a specific location in the environment [14], we would like to understand how continuously changing vowel dimensions F1 and F2 may be related to brain signals. Is this a case of attractor dynamics? Are these analog-to-digital, i.e. continuous-to-categorical, computations influenced by how different vowels are encoded in the long-term memories of speakers of different languages, which vary significantly in where they “put their vowels” on such a manifold? How do we navigate the vowel trajectories in the continuous space of vowels?

### 1.2. Grid cells

In rodents, the localization of the animal in a box, as used in several experimental paradigms, is expressed at the neural level in a few distinct cell populations in the hippocampus and adjacent cortical regions. Among them, grid cells have been an amazing discovery, as they collectively encode position by being activated at the nodes of a hexagonal grid specific to each cell [15], but sharing its orientation and grid spacing with neighboring cells. The ensemble of hexagonally symmetric maps expressed by different cells appears to be stapled together as collection of multiple sheets, which is applied to any environment the animal finds itself in [16]. Since the nodes at which different cells are activated are spread evenly, at least in a flat open arena, while the three axes at 60° of each other are common to all nearby cells, a linear trajectory through the arena is expected to elicit a summed population activity which depends only on its orientation relative to the three axes, hence with a 6-fold symmetry, and does not depend on its absolute location in the arena. This expectation was the basis for the approach used to investigate whether grid cells may be present in the human brain.

**Figure 2.**
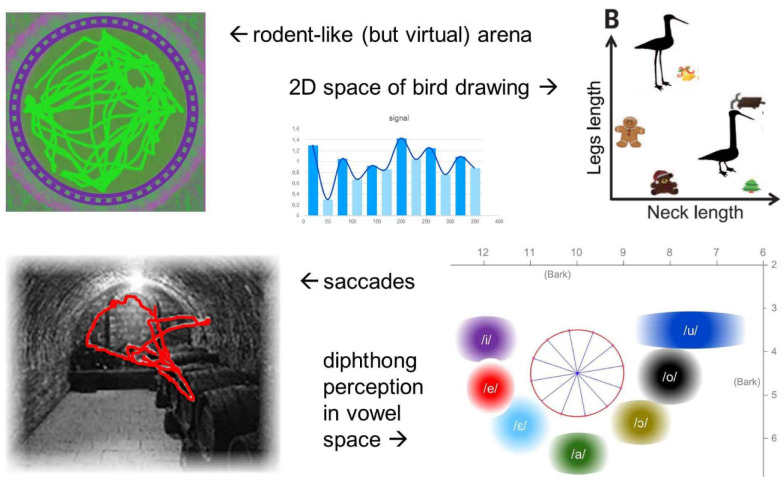
Searching for grid-like signals in mass neural activity in the human brain. Whether with fMRI (top row) or electrophysiological measures (bottom), a common approach (top center) has been to contrast the signal correlated with trajectories at φ+n×60° with those at φ+n×30°, where φ is chosen such as to maximize the difference. Trajectories can be in physical (left) or more abstract spaces (right). Illustrations redrawn from those in Refs. [17],[18],[19].

Doeller and colleagues discovered a signal suggestive of the presence of grid-cell-like representations in humans using functional magnetic resonance imaging (fMRI) [3]. They recorded the BOLD activity of human participants while they explored a virtual environment. The task involved finding and replacing items in their correct locations. The authors hypothesized that if grid-cell firing is present, there should be an effect of running direction with 6-fold rotational symmetry, reflecting the differences between running aligned (larger signal) or misaligned (smaller signal) to the grid orientation, and assuming the orientation of grid cells to be similar across large regions of the entorhinal cortex (EC), one should be able to detect this effect from bulk population activity, as a proxy for recording grid cells. To test whether fMRI signal is modulated by navigation direction with a 60° periodicity, the data was divided into two, and from the first half, the participant-specific grid orientation in EC was obtained. The modulation of the sinusoidal regressor aligned with it was then derived from the second half of the data. A 60° modulation was most significant in the right EC, but surprisingly, and different from what is observed in rodents, this modulation was present in a variety of other additional regions including posterior parietal, lateral temporal, medial prefrontal and medial parietal cortices. They carried out further controls with e.g. 45°(8-fold) and 90°(4-fold) symmetry, which were not significant. The average fMRI signal relative to the baseline of the voxels in the right EC that displayed 6-fold rotational symmetry is shown in the top panel of Figure 2.

Recently, further supporting evidence has been revealed by two independent studies with different recording techniques. In one study, the participants viewed pictures containing both indoor and outdoor scenes. The authors investigated the basis of grid-like coding during free viewing of natural scenes by combined recording of magnetoencephalography (MEG) and eye-tracking activity from healthy humans, and by simultaneously recording intracranial electroencephalography (iEEG) and eye-tracking data with depth electrodes in the entorhinal cortex of one epilepsy patient [17]. In the second study, they recorded only iEEG activity from neurosurgical patients as they performed a virtual navigation task [18]. Analyzing different frequency bands, both studies showed hexadirectional modulation of oscillatory power.

### 1.3. From spatial navigation to generalized navigation

The experiments conducted by Doeller et al are suggestive of the presence of grid-like cells in humans [3]. An important question that arises is whether their functional contribution is restricted only to the representation of physical spaces or whether such neural hardware can represent more abstract spaces. One experiment which has explored this possibility is the study by Constaninescu et al. (2016). In their study, they constructed such a “conceptual space”, or a bird space, that could be organized into a mental map, arguably similar to the way the concepts are represented in the brain [4]. Participants were trained to navigate this space by showing them trajectories in which a bird was continuously morphed from one shape to another. The two dimensions were the length of legs and length of neck of a bird, and the ratio of the former over the latter defined the direction of morphing. The morph trajectories would sometimes pass through locations in the space which were arbitrarily associated with Christmas symbols, and the participants had to learn the association between the bird shapes and the symbols. During the fMRI scanning, on each trial, participants watched an initial bird morph in a random direction for a second, then they were instructed to imagine the morphing to continue in that same direction for a further four seconds. To complete the trial, participants had to choose the Christmas symbol that would have been encountered along the morph trajectory.

Similar to what had previously been observed in the virtual navigation experiments, the fMRI signal recorded during this task was shown to be modulated by the morphing direction with a 6-fold periodicity. Moreover, this 6-fold periodic signal was found in a network of regions that overlapped with regions activated during spatial navigation. It seems then that the grid cell system may be able to encode, in addition to physical space, more abstract spaces, even in an artificial task of the type designed by Constantinescu et al. Can it be that such a code, beyond representing physical space, is a way of representing two-dimensional abstract spaces in general?

Can this grid-like code extend to vowels, as we navigate their naturally continuous and abstract quasi-two-dimensional space every few tens of milliseconds, speaking and listening to others? Comparing the position of standard vowels across languages, we notice that while in Italian, as shown in Figure 1, they are sitting close to the edges, leaving the central region empty, in many languages, including e.g. British English, they “tile” also the inner space. This crowding of familiar acoustic objects likely distorts local geometry, more than in languages with an empty center. To approximate a large flat arena we focus therefore on Italian listeners and attempt, through an extensive training procedure, to get subjects to “iron out” their perception, to be flat in Bark coordinates. We then aimed to search for hexagonal modulation as a function of the direction of change of the first two formants, over two hundred milliseconds, with EEG, which has the necessary temporal resolution.

## 2. Materials and method

### 2.1. Experimental design

#### 2.1.1. Stimuli

**Figure 3.**
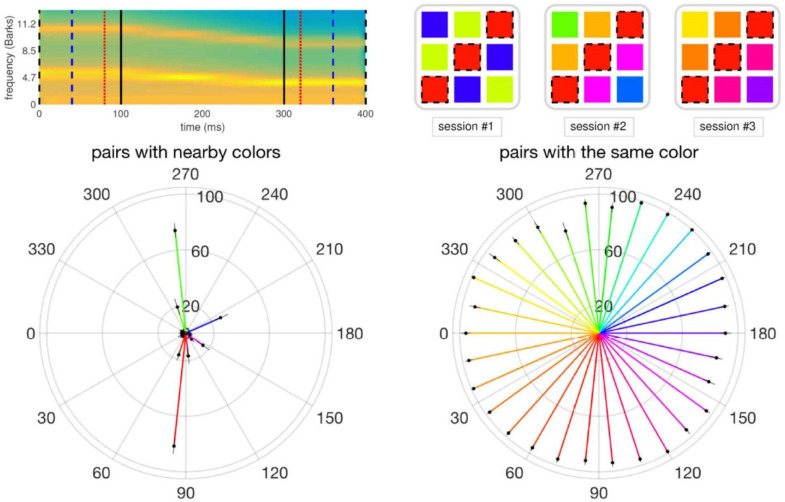
Trajectories, colors and hue discrimination results. (Top left) sample stimulus, sounding roughly like /εu/. (Top right) Progressively more difficult trajectory discrimination testing in the 3 sessions (blocks) of the EEG experiment. (Bottom) results of the preliminary hue discrimination experiment; percent of “same” responses is indicated by the length of each arrow.

In order to reveal a potential 6-fold modulation across directions in vowel trajectories, and compare it, e.g. with a 5-fold one, which we wanted to include as a control, we have created, using the Klatt speech synthesizer [20], 30 artificial quasi-diphthongs as vowel trajectories at 30 equi-spaced angles in the “empty” central circle centered at (10, 4.5) Bark, with radius of 1 Bark (see Figure 1). As noted in the Introduction, the Bark scale is an auditory scale where equal steps in formants correspond to approximately equal steps of perceptual distance; it is roughly linear below 1000 Hz and becomes more logarithmic above. We convert a frequency *f* in Hz to Bark according to the formula in Ref. [19]

$$Bark\left(f\right)=13\text{atan}\left(0.00076f\right)+3.5\text{atan}\left({\left(\frac{f}{7500}\right)}^{2}\right)$$ (1)

and invert Eq (1) numerically to go back from Bark to Hz. Each of the trajectories has steady start and end parts, meaning that during these portions of the stimulus, both of duration Δ, its F1 and F2 values are constant, and one dynamic part in the middle during which the stimulus evolves from the starting phase to the end phase over 200 milliseconds (see Figure 3, top left). Instead of the 12 trajectories shown in Figures 1 and 2, we used 30 in the experiment simply to sample the angle φ more densely, at 12° intervals.

#### 2.1.2. Participants and preliminary hue discrimination task

22 native non-bilingual Italian speakers (local students) with no or very limited second language skills participated in the EEG experiment. Since to perform the task in the experiment, participants had to learn how “to navigate in the central region”, i.e., to associate each trajectory to a distinct color hue, they were first given a simple psychophysical shade discrimination task, which helped them acquire awareness of the fine differences between the hues to be later associated with the vowel trajectories. At each trial, participants first saw a fixation cross on the screen for 2 seconds, then a pair of squares with the same color or two different colors separated by 12° were presented. Participants had 2 seconds to respond if they perceived the hues of the two squares as identical. A new trial started after the 2 seconds limit unless a “same” responses had already been made. There were 30 × 2 (60) trials in one session and 60 × 3 (180) trials in total. Half of the trials included the pairs with the identical color, and the other half included pairs with the neighboring colors. Participants had a 40 seconds long break in between every two sessions. We emphasize that, just like the 30 trajectories did not correspond to real diphthongs, and were purely defined by the metric of the central wheel, so the hues were artificially paired to the trajectory, with a random rotation for each participant, and had nothing to do with the color coding in Figures 1 and 2.

#### 2.1.3. EEG behavioral procedure

The EEG experiment included 3 blocks of *training* and *testing* session pairs. After every training session, during which the participants had to learn to perceive each trajectory and associate it to a distinct color hue (e.g. the red square in Figure 3, top right), they were tested on their newly acquired skill in the test session. Participants had a 40 seconds long break in between every two blocks.

Throughout the three *training* sessions, participants experienced 10 × 30 (300) trajectories in total; half of which were long trajectories that have Δ = 100 ms long steady parts (i.e., 400 ms in total length), and half were intermediate trajectories that have Δ = 60 ms long steady parts (i.e., 320 ms in total length). The 10 presentations of 30 trajectories were distributed unevenly across the three training sessions, such that the first one provided the most extended exposure to the participants, to facilitate their initial learning. There were 5 × 30 (150) trajectories in the first training session; of which 3 × 30 were of the long and 2 × 30 were of the intermediate type. Subsequent training sessions were shorter, to help participants sustain their attention. Thus, the second training session had 3 × 30 trajectories; 30 of which were long, and 2 × 30 intermediate ones. The last was the shortest training session, with 2 × 30 trajectories; 30 long and 30 intermediate. Along the 2D vowel space, the participants were guided during training by being shown a color square in a hue extracted from a continuous color wheel (Figure 3), which was randomly rotated for each participant, so that sound (trajectory)-color associations could be established, and they would be different across participants. Every training trial started with 1.5 seconds of silence, followed by the presentation of the sound trajectory. 200 ms after the end of the sound, the associated color square appeared and stayed on center of the screen for a further second.

In order to have them engaged in a mental exploration of a wider vowel space, they were *tested* on their discrimination of short trajectories (i.e., Δ = 20 ms, or 240 ms in total length), for which they were expected to extrapolate across the steady start and end parts, which they heard for only 20 ms each, similar to the task the participants did in navigating the bird space, in which they had to imagine the morphing to be continued in that same direction of an initial bird morph they watched [4]. Every testing trial also started with 1.5 seconds of silence, followed by the presentation of the short sound trajectory. 200 ms after the end of the sound, the response screen appeared with three color options one of which was always the correct answer. The proximity of the choices increased over three different testing sessions (first at 0° ± 120°, then at 0° ± 72°, finally at 0° ± 48° of each other, with the correct choice equally likely to be in each of the relative positions, left, central or right). This way the participants could refine their associations and become increasingly precise in assessing trajectory direction. All response choices associated with one of the sounds in different sessions are illustrated in Figure 3, top right. One test session had 3 × 30 (90) trials, and the performance on the wheel was assessed over 3 × 90 (270) trials.

The participants could evaluate their learning progress with the quiz questions they were asked during training. After every 30 trials, the training was interrupted with 3 questions that resembled the test trials with a significant difference, i.e. that not short, but long (400 ms) sounds were presented. There was 1 quiz question per trajectory, i.e., 30 questions in total. The color options of the quiz trials were as close to each other as the ones given in the following test session so that the participants were prepared to experience the same level of difficulty during the test. When the participants made a choice, they were given feedback as correct or wrong, but they were not told the right option in case of mistakes.

### 2.2. EEG analysis

#### 2.2.1. EEG data collection and preprocessing

EEG data were collected in a sound-proof booth. The stimuli were presented at a comfortable and constant volume from headphones. The brain activity was recorded with a 64 channel BioSemiActiveTwo system (BioSemi Inc., Amsterdam, Netherlands) at a sampling rate of 1024 Hz. A Common Mode Sense (CMS) active electrode was used as the reference, and a Driven Right Leg (DRL) passive electrode was used as the ground. Two external electrodes placed on the right and left of the outer canthi, and one external electrode placed under one eye were used to obtain horizontal and vertical electrooculograms (EOG). Two additional electrodes were placed on the left and right mastoids. Individual electrode offsets were kept between 30 µV. Participants were requested to minimize movement throughout the experiment except when they had a break. EEG data preprocessing was performed with EEGLAB toolbox [21]. Offline data was imported by reference to the average of the mastoids as common reference averaging is not preferred for studies of auditory evoked potentials [22], and then band-pass filtered (0.1–30 Hz). Following the segmentation of the EEG data into 625 ms long epochs starting at around 200 ms before stimulus onset and 185 ms after stimulus offset, bad channels were discarded using the EEGLAB pop_rejchan function [21]. Trials containing extreme values (200 µV) were eliminated. On average 7% of data was removed for each subject. Independent Component Analysis (ICA) was used to remove eye blinks and muscle artifacts [21],[23]. At this point, trials with correct and wrong answers were separated.

There were on average 5.36 trials per subject per trajectory (out of 9) in the final data set of correct responses. For the clustering and ERP analysis (see below), the data was divided into the desired conditions, and then it was pruned by randomly discarding trials to ensure the same amount of trials per condition. Finally, missing channels, fewer than 10% of all channels, were interpolated, which was followed by a baseline correction with a reference interval of 200 ms before stimulus onset. The resulting dataset of each condition had the data of each participant averaged over trials.

#### 2.2.2. Reduced description of the ERP

For every subject, trajectory, and electrode, we first averaged the EEG data over trials in which participants responded correctly. Two participants who had no correct trial on one trajectory, after preprocessing to remove suspected artifacts, and one with no correct trial on two trajectories were discarded in this analysis. Therefore, the reported results are from the data of the remaining 19 participants.

Based on a preliminary review of the data, we have focused on the EEG signal recorded by the electrodes in three different regions, each including up to 10 electrodes. We refer to them from now on as right parietal, left parietal, and fronto-central regions.

Taking the average evoked response potential (ERP) across participants and trajectories along the duration of a trial, from 200 ms before sound onset to 190 ms after offset, reveals a similar EEG time course in each of the three regions (albeit with larger amplitude in the fronto-central region). One can identify a maximum ERP around 30 ms (which we denote as P50), a minimum around 85 ms (N100), a maximum around 190 ms (P200), and finally a maximum around 340 ms (P350, presumably related to the stimulus offset which in our paradigm was at 240 ms), as will be shown below (see, in the example of Figure 6 (left), the vertical dashed lines in light grey).

We take these 4 extremum values to provide a reduced description of the EEG signal. However, to suppress inter-subject and inter-trial variability, we focus not on the values themselves but on the 4 differences: [P50–N100], [P200–N100], [P350–N100], and the temporal average of the EEG signal between onset of stimuli (0 ms) and end of trial (425 ms) minus N100, [AVG–N100]. Therefore, what will be referred to as the ERPs, at their 4 distinct “time points”, in the following, will be the 4-component vectors arranged in the order [P50–N100], [AVG–N100], [P200–N100], [P350–N100].

#### 2.2.3. Fourier analysis along the wheel

For a Fourier decomposition along the wheel of the ERPs (see Results), after taking averages over trials, we z-scored the data along the wheel, in order to normalize across participants. The z-score for each participant at each angle is calculated as

$$z\left(\theta \right)=z\left(i*12{}^{\circ}\right)=\frac{r\left(i\right)-\;<r>}{\sigma }$$ (2)

where *i* = θ/12° in Eq (2) is the trajectory index around the wheel, <*r*> is the average across trajectories, and σ is the standard deviation of *r(i)* along the wheel.

As the aim of the Fourier decomposition is to find the contribution of each Fourier component irrespective of its phase, we use it in the form of Eq (3)

$$z\left(\theta \right)=\frac{C_{0}}{2}+\Sigma_{n=1}^{15}\left[c_{n}\cos\left(n\theta -n\varphi_{n}\right)\right]$$ (3)

#### 2.2.4. Principal Component Analysis

In the principal component analysis (PCA), in order to study the variation of the ERPs along the wheel (see Results), we did not z-score them but rather, for each individual participant, we subtracted from the trial-average ERPs for each trajectory, their means across trajectories. Afterwards, to suppress the variability due to the limited number of trials per trajectory, we smoothed the ERPs with a quasi-Gaussian approximation, yielding a “signal”, *S*, which is calculated for every trajectory as

S(i)=0.05r(i−2)+0.25r(i−1)+0.40r(i)+0.25r(i+1)+0.05r(i+2) (4)

where *r*(*i*) is the ERP value for the *i*-th trajectory. Note that we did not apply the Gaussian smoothing of Eq (4) to the Fourier analysis.

By averaging over a cluster of electrodes among 10 in each of the 3 regions (left parietal, right parietal, and fronto-central) there would be 12 EEG signals as a function of trajectory along the wheel (each set of electrodes contributes 4 ERP differences corresponding to the four time points) which can be expressed by the 12-component vector *S_k_(i)* in Eq (5), where *k = 4(l-1) + j* labels the signal (still a function of trajectory) obtained in region *l* at the *j*^th^ time point:

$$S=\left[\begin{array}{c} S1\\ S2\\ S3\\ S4\\ .\\ .\\ S12 \end{array}\right]$$ (5)

To better describe the variation of the ERPs along the wheel, we identify the linear transformation of these 12 components that diagonalizes the 12-by-12 covariance matrix whose (n,m) entry is

$$M_{n,m}=cov\left(Sn,Sm\right)=\frac{1}{30}\sum_{i=1}^{30}\left(Sn,i-\;<Sn>)(Sm,i-<Sm>\right)$$ (6)

where <*Sm*> and <*Sn*> in Eq (6) are the *m*^th^ and *n*^th^ entries in the vector *S* averaged across trajectories, and order the eigenvectors in terms of decreasing eigenvalues.

In order to test whether the PCA results remain similar regardless of the number of electrodes considered in each cluster from which we took the average, we compared the eigenvalues obtained with different cluster size. We also looked at the Pearson correlation between eigenvectors components (see Results), defined between two variables *X,Y* as

$$\rho_{X,Y\;}=\;\frac{Cov\;(X,Y)}{\sigma_{x}\sigma_{y}}$$ (7)

where Cov and in Eq (7) refer to covariance and standard deviation respectively.

It should be noted that, only for the PCA analysis, the time points for each subject are calculated separately, by searching for extrema in a 20-msec window around the time points identified for the subject-averaged ERPs. All error bars in the figures and tables are due to inter-subject variability, and represent standard errors of the mean, calculated as the ratio of the standard deviation by the square root of the number of subjects.

## 3. Results

### 3.1. Behavioral results

The results of the shade discrimination experiment, shown in Figure 3 (bottom), indicate that the participants had no major issues in discriminating among 30 colors, which otherwise could have created a problem for their sound trajectory discrimination. It is true that they are more challenged with perceiving differences between two shades of red and two shades of green, however these were 12° apart (whereas for sound trajectory classification they were at least 48° apart) and also, since the color wheel was rotated across participants, colors were linked to random trajectories.

**Figure 4.**
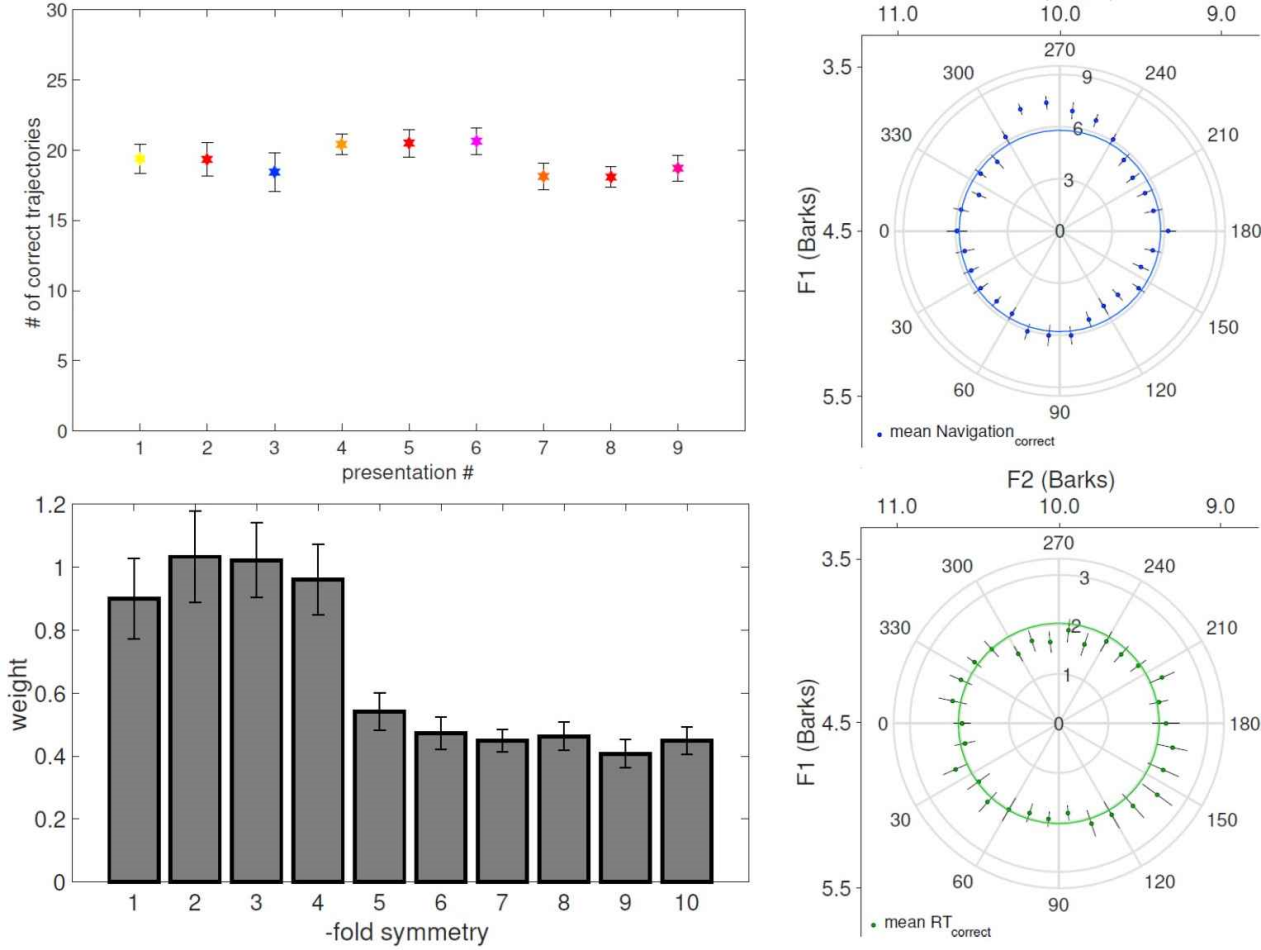
Behavior in the EEG experiment. Performance was relatively stable around 65% correct along the 3 sessions, with gradual learning offsetting increasing difficulty (top left). Also as a function of θ, performance was only slightly better in the direction between /i/ and /u/ (top right), with a correspondingly shorter reaction time (bottom right). The Fourier decomposition did not reveal any dominant component in the behavior (bottom left).

The performance on the main experiment was roughly constant across 9 presentations of 30 trajectories in 3 testing sessions, as desired (Figure 4, top left). Although the task became more difficult at each session, gradual training helped participants maintain the level of navigation they could achieve. Gradual learning is reflected in the slight increase in the second session in which the second three presentations (presentation # 4,5, and 6) occurred. On the other hand, a slight decrease in the last three presentations (presentation # 7,8, and 9) could likely be explained by the difficulty of the last session due to the increased proximity of the choice options, and some mental fatigue by the participants.

Equally good discrimination of all 30 trajectories was aimed for, in order to reveal a possible grid-like representation of trajectories along the vowel wheel in the middle of the vowel space. The top right plot in Figure 4 shows that this is approximately achieved, as reflected in the quasi-circular proportion of correct responses. The inner circle has the radius of the average correct response across 30 trajectories. The responses diverge from the circle especially in the region between 240° and 300° degrees. This region is where the initial 20 ms of the auditory stimulus has the formants in between the vowels /i/ and /u/, where there are no other standard vowel categories in Italian, whereas the final 20 ms would correspond, if extrapolated outside the wheel, to the Italian standard vowel /a/. Better performance in those trajectories is accompanied by shorter reaction times, as shown in the bottom right plot in Figure 4, in which the inner green circle denotes the average reaction time of correct behavior. Interestingly, the better performance is not entirely symmetric, i.e., the trajectories, which end in that region and start from the opposite bottom part between 60° and 120° degrees, are not that well discriminated. If we look at the correct responses session by session, we observe no dramatic difference in the behavior either (not shown). Trajectories between 270° and 300° degrees are identified better and in a shorter time in all sessions, but the increased performance between 240° and 270° is mostly specific to the first session, where the two wrong choices are easier to discard. It is also observed that it takes more time to recognize the trajectories starting from the region between 150° and 210° degrees, and the correct responses are particularly low, there, in the first session. That portion of the wheel is close to space where vowels /o/ and /ɔ/ are.

We have first checked for any periodicity displayed in the behavior, to make sure a possible hexagonal or any other symmetry in neural activity is not due to the way the perceptual wheel is deformed. Utilizing the Fourier expansion described in Methods, we show in Figure 4 (bottom left) the weight *c_n_* of each cosine, which we call “fold”, up to 10, with the phase *φ_n_* chosen in each case so as to eliminate the sine component. The first four components have higher weight, but none of them significantly greater than the others. We carried out a similar control test by fixing the color location, and keeping now the phase *φ_n_* constant across subjects, since rotating the responses for each subject fixing the color location would yield the same coefficients as for diphthong trajectories, but with different participant-specific phases. In relation to color, too, we found no specific symmetry that better approximates the responses.

### 3.2. EEG results

We employed nonparametric clustering analysis as a method of systematic exploration of the neural correlates of navigation along the wheel, as there are many time points, electrodes, and possible combinations of trajectories. We refer to Refs. [24],[25] for the details of the method, that we applied in conjunction with Fourier analysis, and with Principal Component Analysis, described under Methods.

#### 3.2.1. Slicing and rotating the vowel wheel

As a preliminary investigation of periodicity in the neural data, and to understand the changes in the EEG signal as a function of F1 and F2, for the values of the periodicity *n* between 1 and 6, we divided the trajectories into two conditions, because the way our clustering method is implemented works only with 2 conditions. For example, for 1-fold symmetry, the data of 30 trajectories are divided into two sets each containing 15 trajectories, and clustering analysis is applied. This is repeated for 15 rotations of the wheel to check for every possible combination of the trajectories in 2 main clusters of directions. In our example, what the clustering analysis would help us to find is if there is one particular direction that the neural activity is significantly sensitive to, so that there is a significant difference reflected in the amplitude of the neural activity in the processing of the wide band trajectories starting and ending in opposite directions, and by which angle that divides the vowel plane this difference is maximized, in addition to when and in which region it occurs in the brain. The same reasoning is used to perform the analysis with larger values *n* of the periodicity. This is somewhat an indirect analysis of the periodicity, but useful to get an idea about the signal, as there are many possibilities to check.

We inspected all the clusters obtained with an arbitrary threshold of p < 0.3. The time window of the analysis was from the onset of the stimulus till 180 ms after end of it, but we mainly focused on the clusters that show some difference around 100 ms and 200 ms, as the literature [26],[27] and our own earlier finding in a separate study suggested a modulation of the activity based on the first two formants around these two time points, and on the clusters that last longer than 5 ms. We were not able to find such “meaningful” clusters for many of the conditions we explored. The activity of one of the promising clusters (t = −113.26, p = 0.2389) we observed is shown in Figure 6 (left) for the 6-fold symmetry condition, as illustrated on the small wheel overlaid on the same figure. A maximal difference between two conditions, which are blue and red trajectories at every 5th direction as depicted on the wheel, is reached around 85 ms stimulus onset latency over the 10 right occipito-parietal electrodes shown in the topography map of the difference. In the Fourier analysis, we used these 10 electrodes to search for periodicity directly. As a control, we also included 10 left occipito-parietal electrodes, that locate on the mirror symmetrical positions of the ones above, and a set of 10 fronto-central electrodes, a region of interest in the studies of auditory processing [28],[29],[22].

#### 3.2.2. Fourier analysis

**Figure 5.**
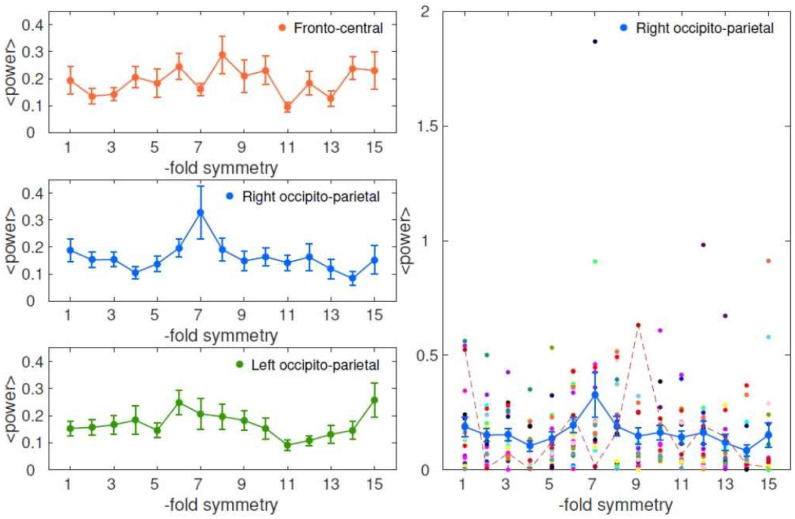
Mean power spectrum of the EEG signal in three different regions. (Left) The EEG signal computed as the difference between P50 and N100 does not display a particular periodicity in any 3 regions of interest, as seen from the average power spectrum. (Right) 10 electrodes in the right occipito-parietal region show a greater 7-fold symmetry compared to the other components, as also seen in the middle figure of the left panel, but the individual data points of 19 participants in colored circles show that it is mostly due to 2 subjects with high 7-fold coefficient, hence the large error bars demoting SEM across participants. The dashed lines show the power spectrum of the EEG signal of an exemplar subject that has low 7th component.

Is there a rotational symmetry in the brain representation of the vowel wheel, similar to the hexagonal symmetry reported for bird drawings that vary in two dimensions? If there is such a symmetry, the Fourier decomposition of the EEG signal on the wheel should show, for at least some specific time points and some electrodes, a dominant contribution by one of the components, and possibly its harmonics, which is expressed in a large amplitude across subjects, although the phase might vary from subject to subject. In the left panel of Figure 5, for the 3 different regions mentioned above, we show the power spectrum, i.e. the square magnitude of the Fourier coefficients averaged across subjects, where smaller colored circles at each fold denote the data of individual participants. The signal of each subject is taken as the difference between P50 and N100. We see that the 6-fold component is indeed somewhat stronger than the others (except the 15-fold) in the left parietal region, but in the fronto-central region it is the 8-fold component which is stronger, and in the right parietal region the 7-fold, in fact apparently much stronger than the others. At a closer look, however, we see that individual participants present widely diverse Fourier coefficients, and the 7-fold “dominance” in right parietal electrodes is due solely to the contribution of a couple of subjects (Figure 5 right, note the black line, indicating a participant with yet another stronger component, the 9-fold one).

In the same manner, not to limit our analysis to a specific time point such as [P50–N100] as in Figure 5, we extended our analysis to the whole time course of one epoch, that starts 200 ms before the sound onset and ends 185 ms after the end of the sound, by subtracting the N100 peak. However, we did not observe periodic behavior at other different time points of the signal either. We observe a 7-th component at around 50 ms, too soon and too short to be considered as a real effect of the navigation. We also looked for the periodicity in another set of electrodes that are between left and right occipito-parietal regions at around 240 ms, due to our observation of another borderline cluster (p > 0.05), but also there, we could not find a significant Fourier component.

#### 3.2.3. Diphthongs vs. vowels

We have placed the wheel in the center of the vowel space, where there are no standard vowel categories in Italian. Vowels along the wheel may be thought to act like landmarks that guide navigation in spatial environments, and through extensive training, the mental representation of this central space might become flattened (in Bark coordinates). Do we see any interesting modulation of the neural signal according to the first two formant frequencies along the wheel, in this empty center?

**Figure 6.**
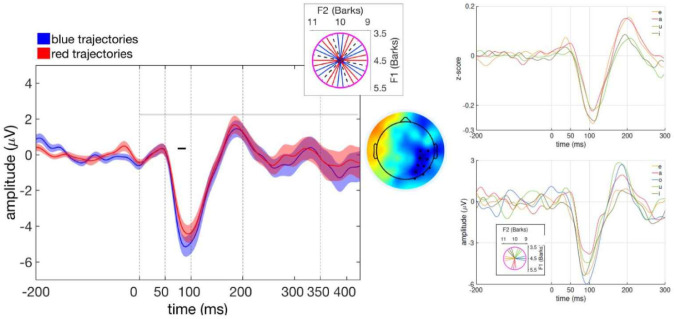
ERP time course shows subtle differences. (Left) Grand average of two set of trajectories at every 5th direction. Time 0 indicates the onset of the stimulus. Error bands denote the SEM across participants. The horizontal black line delimits the time range of the significant cluster (p > 0.05) between the 2 conditions, shown in the topography. The top inset shows the trajectories in 2 conditions at every 5 directions. (Right) Average time course to consonant-vowel syllables ending in one of 4 vowels /a/, /e/, /u/, /i/, from a separate experiment, top, and to 5 clusters of trajectories starting close to 5 of the 7 Italian standard vowels: /e/ /a/ /i/ /u/ and /o/ (bottom). Note the mismatch in the results, possibly due to the difference between single vowels and artificial quasi-diphthongs.

Outside of the center, the evoked potentials recorded in a separate experiment (to be reported elsewhere) with native Italian listeners to (American) English consonant-vowel pairs, which end with one of the four standard vowels /e/ /a/ /i/ /u/, suggest two levels of processing of the formant frequencies, which occur at two different time points. As seen in Figure 6, top right, around 100 ms (N100), the neural trajectories in the other experiment are clustered into two groups based on the degree of frontness, or backness of the ending vowels. The EEG signal of the front vowels /e/ and /i/ shows a greater negative deflection than the back vowels /a/ and /u/. In the same figure, we see, around 200 ms (P200) after syllable onset, the grouping of the neural signal is based on the degree of openness, and that the amplitude of the positive deflection for the open vowels /e/ and /a/ is greater than that for the close vowels /i/ and /u/. Is such F2 and F1 processing as reflected by the N100 and P200 components also valid for the neural processing of the empty central region, in the present experiment?

In order to see if such an imprint of formant processing is also observed for the neural correlates of our quasi-diphthongs, we looked at the EEG signal of the trajectories that belong to different portions of the wheel. We grouped 3 consecutive trajectories on the wheel into 5 groups of different diphthongs, (not all equi-spaced), according to the proximity of their starting direction to 5 standard vowels, /e/ /a/ /i/ /u/ and /o/ as in the inset of the bottom right plot in Figure 6. That plot shows that at around 100 ms, although the processing of the steady initial components of the four diphthongs, /e/ /a/ /i/ and /u/, is reminiscent of the processing of the vowels of the outer region, the neural signal of the steady part that starts as /o/ casts a doubt on the model which assumes F2 processing at N100. Given our observations on the vowel processing in the perimeter of the vowel space, we would expect the neural correlates of /o/ to be similar to those of /a/ and /u/, as it is a back vowel. However, we should also remember that what participants hear at 100 ms in the two experiments is not the same. In the other experiment, with CV syllables, what they heard was a steady vowel (or a transition into it from a consonant). That is different from what they hear in the present experiment we are discussing. In addition to the 20 ms long steady portion, there have been the initial 80 ms of the long dynamic part of the acoustic signal that has F1 and/or F2 changing in the same or opposite directions (Figure 3). It is puzzling to see that although /e/ and /o/ start from opposite ends of the F2 axis, the difference between the EEG amplitudes of their perception is similar, but of opposite sign, to the difference between the perception of /i/ and /u/ trajectories that have closer second formants. Also puzzling, the difference between the amplitudes of the perception of /i/ and /e/ is much smaller than the difference between /o/ and /u/ even if the F2 distance between /e/ and /i/ is similar, in both cases small, as the one between /o/ and /u/. Moreover, at P200, there is no corresponding clustering between the two panels of Figure 6 right.

Looking at the bottom plot in the same figure, we see that the trajectories that start as /o/ or /u/ have similar amplitudes. Likewise, the trajectories that start as /e/ or /i/ have also similar amplitudes. In between these two clusters, there are the trajectories that start as /a/. Is this reflecting an F2 processing, which we observed 100 ms earlier with steady vowels? Again, by 200 ms, what participants hear is a 20 ms steady part and a longer portion of the dynamic of the acoustic trajectory. For example, for /o/ and /e/ trajectories, the participants hear trajectories with the same mean F1 and F2 for 160 ms but changing in two opposite directions. However, the difference between their signal is even amplified compared to their difference at 100 ms.

What is the difference between the two neural imprints in the center and outside the center due to? Can it be because the sounds are squeezed in a small space at the center, too close to each other, with the same mean formant frequency? Can it be because they are not stable in time and what N100 and P200 reflect includes the dynamic part of the acoustic signal?

#### 3.2.4. Principal component analysis

The patterns at the 4 different time points we have chosen (see Methods) indicate that ERP variation along the wheel is not the same across time. Therefore, with PCA we aimed to characterize the main spatio-temporal ERP features as it varies along the wheel. We studied how this variability is expressed at the four chosen time points, considering a number of options in terms of the spatial distribution of the EEG signals to be taken into account. First, we performed PCA for the 1 by 4 vector corresponding to the four time points obtained by averaging the signal across all 30 electrodes in three regions, i.e., by disregarding their spatial (scalp) distribution.

**Table 1.** Eigenvalues and eigenvectors obtained by disregarding spatial features.

Eigenvalues	0.862	0.149	0.065	0.006
Eigenvectors	1st	2nd	3rd	4th
[P50–N100]	0.28	0.15	0.94	0.07
[AVG–N100]	0.34	0.14	−0.05	−0.92
[P200–N100]	0.16	0.93	−0.21	0.21
[P350–N100]	0.88	−0.27	−0.24	0.29

As reported in Table 1, the first eigenvalue contributes 79% of the variance of the ERP variation along the wheel, and its eigenvector receives the main contribution from the [P350–N100] component. In turn, the second eigenvector is mostly correlated with the [P200–N100], the third with the [P50–N100] and the fourth with the [AVG–N100] component, but their amplitudes are increasingly minimal. This suggests that, beyond the main effect expressed by the first eigenvector, other significant effects, if they exist, might be intrinsic to the spatial scalp distribution, and remain hidden in this non-spatial analysis.

**Figure 7.**
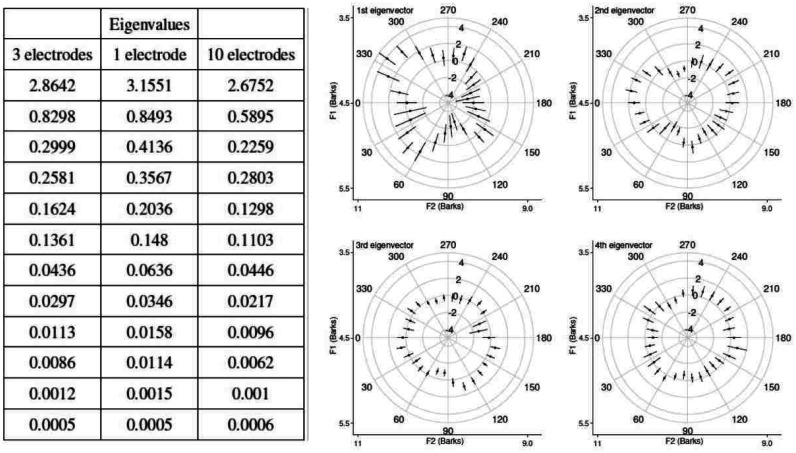
Dominance of the first few eigenmodes. The table (left) shows all 12 eigenvalues, which depend mildly on the number of electrodes averaged in each of the 3 regions. The first 4 eigenvectors, extracted by averaging 3 electrodes per region, are shown on the wheel, on the right.

In order to test this hypothesis, we separate the different regions and first consider 3 electrodes per region, to give a 12-component ERP vector, see Methods. Again, the first eigenvalue (Figure 7, left) accounts for the most of variance (61%). There is a sharp decrease in the following eigenvalues, which account for 0.17, 0.06, and 0.05 of the variance, respectively. After the 6th eigenvalue, only 1% of the variance is accounted for by eigenvalues 7–12.

When only 1 electrode is chosen, there is a trade-off between an increase in the first eigenmode and an increase in variance (not shown; so that their ratio slightly decreases with respect to the 3 electrodes case). When all 10 electrodes in a region are taken into account, the first eigenmode is slightly decreased. The very high values of the Pearson correlation between the corresponding eigenvectors obtained with different number of electrodes (above 0.98 for the first, above 0.95 for the second eigenvector; not shown) indicate that the basic results remain the same regardless of number of electrodes per region. We focus therefore on the results obtained with 3 electrodes per region.

The shape of each polar plot in Figure 7 gives us an idea about the modulation of the EEG signal along the wheel, where the limits of each radial axis are adjusted to the mean of the particular weighted amplitude ±5. Since the first eigenmode is the strongest one, i.e. the one that explains the most of the variance in the EEG signal, the signal transformed into the direction of the first eigenvector is more informative about the change in the signal as a function of direction, as seen on the top left polar plot in Figure 7. The first eigenmode is roughly excited along the 4 main diagonal directions, which start between 30° and 60°, between 120° and 150°, between 240° and 270°, but mostly between 300° and 330°, that is the region where the trajectories start close to an /i/, and end close to an /ɔ/ sound.

For the other eigenmodes, the deformations on the wheel appear to occur along fewer directions and, in particular for the second eigenmode, they seem to be restricted to two main directions along F2, and for the third eigenmode, a bulge is mostly in the direction of trajectories starting between 90° and 180°, where sounds start with low F2 and high F1, and end with high F2 and low F1. These second and third eigenmode deformations, shaped by the variance in the data, do not however relate to the performance, which is strongly peaked between 240° and 300° (see Figure 4).

**Figure 8.**
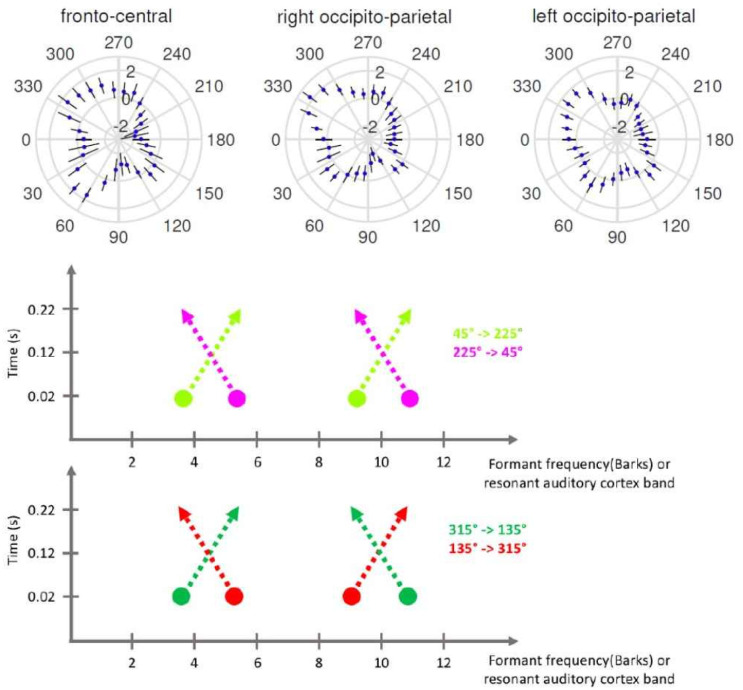
EEG signal of [P350–N100] in three regions. The first eigenvector (see Figure 7) has a shape highly similar to the subtracted EEG signal between P350 and N100, which dominates it in every region, especially in fronto-central and right occipito-parietal regions. In the left occipito-parietal region it has a more inflated and less edgy shape. The diagrams below interpret the shape of the first principal component of the EEG signal in terms to the topographic organization of auditory cortex by spectral frequency (Middle) The first and the second formants of trajectory 45°→225° (in light green) decrease together. For the 180°-opposite trajectory (in pink) both increase in parallel. (Bottom) The trajectory on the most excited diagonal (315°→135° in dark green) has increasing first formant and decreasing second formant. The trajectory 180° across (in red) has the opposite relationship.

When we analyze which time points among the EEG extrema vary the most along the wheel, again we observe that the weight of the fourth component of every region, which corresponds to the difference between the last positive peak and the first negative peak, i.e. [P350–N100], is greater than the difference between other time points and N100, on the first eigenvector. This means that in order to vary the most along the wheel one should move toward the direction which is mainly parallel to [P350–N100]. Therefore, the shape of the principal component, as shown in Figure 7, resembles the shape of the EEG signal of the forth component, as seen in Figure 8 separately for each region. We can conclude that [P350–N100] plays a prominent role in the perception of our trajectories, and perhaps of diphthongs as well.

How is the neural processing around the last positive peak relevant and why does it have a strong dominance compared to the processing at earlier time points? We do not exactly know, but looking at the grand-averages of *correct* and *wrong* trials of all sounds across the 10 electrodes in the fronto-central and right occipito-parietal regions (not shown), we see the difference between the two, although small, is greater in the last positive peak, but also in the last negative peak. Interestingly, while in the fronto-central region, the deflection for wrong trials is larger than for the correct ones, it is reversed from what is exhibited by the right occipitoparietal region. In left occipito-parietal electrodes, this difference occurring at the last positive peak is particularly reduced. Overall, the neural population activity around this last peak might therefore be an important component of the perception of the sound and thus of the final decision.

## 4. Conclusion

It has been tempting to speculate that we represent the overly familiar trapezoidal portion of the (F1, F2) plane somewhere in our brain in similar ways to how we, or at least other mammals, represent familiar 2D spatial environments. A particularly attractive connection [3],[4] could be imagined to the coding expressed by grid cells [15]. The dispersion-focalization theory (DFT) for vowel systems [30], in fact, postulates that vowels tend to place themselves at approximately equal distances from each other on the F1, F2 plane with permissible articulation boundaries [31]. This means that a phonological inventory settles into an optimal arrangement by minimizing an energy function that is a weighted sum of two terms: the first one is the dispersion, that is the maximization of the auditory distances between vowels, and the second one is the local focalization, that is maximization of the importance or perceptual salience of focal vowels. This process of vowels arranging their positions in the space is similar to the self-organization theory of multiple grid fields into a triangular grid pattern with no external drive, but solely due to firing rate adaptation, which is similar to the dispersion term [31]. In a large space with a large number n of vowels they would then be expected to be close to the vertices of a triangular grid, but since n is only 3, 5, 7,11, at most about 15 or so in a constricted space, their position depends heavily on the number. If there is a schwa-like vowel, as there is in English, in order to acquire similar distances from the vowels pushed at the borders it would tend to extend in a third dimension, effectively deforming flat 2D space into something with curvature; but not in languages like Italian without standard vowels in the center. Should we expect, then, the central region to be conceived as a flat arena, in these latter languages like Italian? And would then neural activity demonstrate hexagonal modulation? We know a lot about the neural representations of the flat empty arenas used in many rodent navigation experiment, and linking the two would have allowed us to begin to understand the neural operations our brain carries out on phonemes.

We could not, however, detect a neural signal of phoneme trajectory perception expressing hexagonal symmetry in the center of the 2D vowel plane. One possible reason could be that the vowel space with radius of 1 Bark is not large enough, after all, to develop a grid like representation. Even when it is navigated coast-to-coast, so to speak, in a /io/ diphthong for example, this is done in 100–200 ms in natural speech—possibly too short a time for any unit even inclined to be a grid cell to express multiple fields.

Still, the shape of the first eigenvector suggests that mass activity is not isotropic (rotationally invariant) around the wheel. Is it because neural activity even inside the wheel is sensitive to the presence outside of the standard vowels? That would be a natural outcome of the vowel space being “too small”. But if so, is this influence language dependent, as it should be if it reflects standard vowels which are language dependent? We are testing this hypothesis, that the space is small, hence dominated by the standard vowels, and hence language dependent, in a separate set of experiments. Here, considering the results of the current experiment, one could wonder whether there is a relation of the first eigenmode to existing vowel attractors. We designed the wheel to be in the empty space in the middle among standard Italian vowels. Although the position of standard vowels in any language is highly variable and speaker and context dependent, we can refer e.g. to the authoritative early study by Ferro et al. and express the formant frequencies they cite in Barks [32], and place them around our wheel.

Even taking other more conservative reference values [6], the standard vowels are clearly outside the wheel, as shown in Figure 1. Further, even common Italian diphthongs do not appear to interact much with the wheel. If approximate diphthong trajectories are traced between the standard vowels and the frequency of usage of each diphthong or rather biphone combination is taken from the Phonitalia database [33], one sees that there is very limited “traffic” over our central wheel. Frequent diphthongs, mainly /ia/ or /ja/, /io/, also /jε/, /ju/, /wɔ/, are all outside the wheel. Therefore, we can assume that our wheel trajectories, although diphthong-like, are quite distinct from real common Italian diphthongs and their putative attractors.

What else could the dominating eigenmode be related to? One possibility can be inferred from the fact that the first eigenmode is excited close to the 4 diagonal trajectories on the wheel. The two 45°→225°(/ε/-/a/ to /u/-/w/) and reverse trajectories are those where the first and second formant rise or fall in parallel. In auditory cortex there is tonotopy, with a metric not known in detail, but which we can assume to be close to a Bark scale. Therefore, while the fundamental frequency will be close to constant in time along our trajectories, the first and second resonant frequencies will be two bands of excited neurons moving in parallel one direction or the other (mid row in Figure 8). The most excited diagonal however is the one going from 315° to 135° (/i/-/y/ to /a/-ɔ/), which corresponds to two colliding bands of excitation. Interestingly, the opposite direction is relatively less excited, the red one, corresponding to two diverging bands of excitation. Maybe these physical traits give rise to the eigenmode, which in this case would be language independent, probably. Maybe, instead, to focus on physical traits relevant at the beginning of the auditory pathway could be misleading, if the multidimensional feature space which encodes the acoustic parameters of vowel sounds, and which is beginning to be investigated experimentally [34], is the language-dependent product of a series of transformations in the different auditory cortices [8], In that case, it is likely that even in languages with an “empty center”, the coding of sounds in the center reflects the phonemotopic representations at the periphery. We leave it for a future study to present the wheel test to subject speaking other languages, and so test whether their first eigenvector is different from that for Italians.
